# Association between lifestyle, multiple chronic conditions, mental health status and the severity of lower urinary tract symptoms/benign prostatic hyperplasia in Chinese elderly

**DOI:** 10.3389/fmed.2025.1545344

**Published:** 2025-06-25

**Authors:** Yifan Wu, Yuwei Zhang, Xinwei Liu, Yongneng Huang, Ye Hua, Ninghan Feng

**Affiliations:** ^1^Department of Urology, Jiangnan University Medical Center, Wuxi, China; ^2^Wuxi School of Medicine, Jiangnan University, Wuxi, China; ^3^Medical School of Nantong University, Nantong, China; ^4^Department of Neurology, Jiangnan University Medical Center, Wuxi, China

**Keywords:** lower urinary tract symptoms, benign prostatic hyperplasia, lifestyle, multiple chronic conditions, mental health, geriatric medicine

## Abstract

**Background and objective:**

The etiology and pathogenesis of lower urinary tract symptoms/benign prostatic hyperplasia (LUTS/BPH) are complex, and understanding of factors related to symptom severity can improve the disease prognosis. The aim of this study was to explore the association between LUTS/BPH severity and lifestyle, multiple chronic conditions (MCC), and mental health status in Chinese elderly individuals, and to provide a reference for developing comprehensive intervention measures.

**Methods:**

A total of 806 patients aged 60 and above with LUTS/BPH were divided into mild and moderate-to-severe groups based on IPSS assessment. All participants completed data collection on general demographics, clinical characteristics, lifestyle factors, MCC, and mental health status (including anxiety, depression, and sleep quality). A binary logistic model was employed to investigate the influence of lifestyle, MCC, and mental health status on LUTS/BPH severity.

**Results:**

The moderate-to-severe group had significantly higher rates of unhealthy lifestyle habits, MCC, and mental health problems compared to the mild group. After adjusting for confounders (such as age, disease duration, prostate volume, total prostate-specific antigen, C-reactive protein, post-voided residual urine, urea nitrogen, etc.), current smoking (*OR* = 1.995, 95%CI:1.270–3.134), unhealthy dietary habits (*OR* = 1.590, 95%CI: 1.059–2.386), and lack of active exercise (*OR* = 1.996, 95%CI:1.274–3.127) were positively correlated with the severity of BPH/LUTS. Conversely, the absence of heart disease (*OR* = 0.435, 95%CI:0.268–0.707), normal lipid profile (*OR* = 0.587, 95%CI:0.354–0.973), no diabetes mellitus (*OR* = 0.523, 95%CI:0.312–0.878), no depressive disorder (*OR* = 0.447, 95%CI:0.204–0.979) and no sleep disorder (*OR* = 0.494, 95%CI:0.322–0.758) were significantly negatively correlated with the severity of BPH/LUTS.

**Conclusion:**

The study revealed a strong correlation between the severity of LUTS/BPH and poor lifestyle, multiple chronic diseases, and mental health problems. Therefore, the prevention and treatment of LUTS/BPH should fully consider these factors.

## Introduction

With the development of the social economy and improvement of medical conditions, the aging population has increased dramatically, and health problems related to aging have become increasingly prominent. China is a large country with an aging population, having more than 260 million people over the age of 60. This has led to an increase in the prevalence of chronic diseases and multimorbidity. Benign prostatic hyperplasia (BPH) is a common chronic disease among older men, with an incidence rate of 44.7% among those aged 60–69, which increases significantly with age, reaching 69.2% among those aged 80 and above. This imposes considerable burdens on both families and society (1, 2). It is reported that the global cost of treating BPH is as high as several billion US dollars per year, and China has seen a much higher increase in the disease burden of BPH over the past 30 years than Japan and South Korea. The reason for this may be related to the fact that as the socio-demographic index (SDI) level in China has increased, residents’ dietary patterns and lifestyles have changed, leading to increased exposure of men to the risk factors for BPH (3, 4). BPH is the main cause of lower urinary tract symptoms (LUTS) in elderly men, characterized by urinary obstruction, weak urine stream, incomplete bladder emptying, increased urinary frequency, urgency, and nocturia. As the disease progresses, LUTS problems become more severe, leading to complications such as urinary tract infections, hematuria, renal insufficiency, acute urinary retention (AUR), urinary incontinence, or ureteral reflux, which seriously affect the quality of life of patients and may even threaten their lives. The etiology and pathogenesis of BPH are multifactorial, with age and sex hormones being the primary predisposing factors for its development. Race, unhealthy lifestyle habits, obesity, chronic inflammation, cardiovascular disease, high blood sugar levels, and other common chronic diseases may contribute to the progression of the condition. Furthermore, psychological factors may also be related to the occurrence and progression of BPH/LUTS (5, 6). The current treatment options for BPH primarily consist of watchful waiting, pharmaceutical intervention, and surgical procedures. However, pharmaceutical intervention lacks specificity and is associated with a high recurrence rate. Despite surgery, between 20 and 50% of patients may still require intermittent self-catheterization or permanent indwelling catheterization, or continue to experience persistent LUTS without achieving the desired clinical efficacy (7, 8). McConnell et al. conducted a 4-year observational follow-up study on mild LUTS patients, providing them with routine health education, lifestyle guidance, and regular monitoring. The findings indicated an annual clinical progression rate of 4.5% among patients, with no evidence of acute renal damage resulting from urinary tract obstruction (9). Therefore, the establishment of a hospital-centered and community-based model for the prevention and control of BPH, incorporating targeted modifications to modifiable factors, holds significant importance in effectively delaying the progression of BPH. Currently, the majority of studies in China primarily focus on the relationship between metabolic syndrome and benign prostatic hyperplasia (BPH), or merely examine a restricted number of risk factors among numerous possibilities. There is limited literature addressing the association between lifestyle, multimorbidity, mental health, and the severity of BPH/LUTS. In particular, there is a paucity of research encompassing all three dimensions—lifestyle, multimorbidity, and mental health—within the Chinese population. The prevalence of psychological disorders among patients in general hospitals can exceed 30%, which complicates the treatment of physical diseases. However, non-psychiatrists, particularly surgeons, lack proficiency in identifying psychological issues and awareness of intervention (10–13). The purpose of this study was to investigate the relationship between the severity of BPH/LUTS and the lifestyle, comorbidity and mental health status of the elderly individuals in China, in order to enhance clinicians’ ability to comprehensively assess BPH/LUTS and better tailor individualized intervention strategies for patients. We hypothesized that: ① Unhealthy lifestyles, comorbidities of chronic diseases, and psychological problems are independently associated with the severity of BPH/LUTS; ② The proportion of unhealthy lifestyles, chronic disease comorbidities, and psychological issues among patients with moderate to severe benign prostatic hyperplasia (BPH) is relatively high.

## Materials and methods

### Subjects

This was an observational, cross-sectional study. The inpatients at the Urology Department of Wuxi Second People’s Hospital were selected from October 2023 to April 2024. Inclusion criteria:① Individuals aged 60 years or older; ② Diagnosed with BPH based on prostate biopsy; ③ Experiencing one or more LUTS, such as frequent urination, urgency, dysuria, weak urine flow, increased nocturnal urination, etc.; ④ Received standard treatment for the first time at our hospital; ⑤ Able to cooperate with the completion of relevant laboratory tests, imaging examinations, and psychological health questionnaires. Exclusion criteria: ① Severe untreated systemic diseases or other unstable physical conditions;② Urological malignancies or combined systemic tumors; ③ Serious mental illnesses, severe anxiety or depression disorder and senile dementia; ④ History of previous prostate surgery. All patients and their families were required to sign informed consent. This study was conducted in accordance with the Declaration of Helsinki and approved by the Ethics Committee of Wuxi No.2 People’s Hospital (No:2021-Y-3).

### Data collection and assessments

#### Clinical interviews and examinations

① Socio-demographic data collection: name, age, marital status, education level, smoking and drinking habits, lifestyle habits, etc.; ② clinical data collection: disease course, lower urinary tract symptoms, past medical history, personal history, physical examination, laboratory tests (blood count, urinalysis, C-reactive protein, blood sugar, cholesterol, thyroid function, liver and kidney function, total prostate specific antigen in serum), imaging examinations (Electrocardiogram, B-ultrasound, residual urine determination, prostate volume measurement), biopsy of prostate tissue for histological examination, etc.

#### LUTS assessment

The International Prostate Symptom Score (IPSS) was used to assess the severity of lower urinary tract symptoms (14). The IPSS includes 7 dimensions of symptom problems, including incomplete emptying, frequency, intermittency, urgency, weak stream, straining, and nocturia. Each dimension is scored from 0 to 5 based on the frequency of symptoms. The total score ranges from 0 to 35, with a higher score indicating more severe LUTS. In this study, patients were divided into mild and moderate-to-severe groups based on IPSS scores, with the mild group having an IPSS score of 0–7 and the moderate-to-severe group having an IPSS score of ≥8.

#### Healthy lifestyle assessment

Based on the results of the China Kadoorie Biobank (CKB) study and in conjunction with the American Heart Association (AHA) “ideal cardiovascular health” criteria (15, 16), six lifestyle indicators were selected for analysis, including smoking status, alcohol consumption, dietary patterns, physical activity, body weight, and body fat. Current non-smoking status was defined as never having smoked, occasional smoking, and quitting smoking not due to illness; Non-excessive drinking status was defined as drinking less than 25 g/d per day (converted to pure alcohol); Healthy diet was defined as having a frequency of consuming vegetables, fruits, and soy products at least 4 days per week, consuming fish at least 1 day per week, and consuming red meat less than 7 days per week, with the intake frequency of more than 4 types of food meeting the above standards. Active physical activity was defined as engaging in aerobic activities three or more times a week, each session lasting 30 to 60 min; Healthy weight was defined as a Body Mass Index (BMI) ranging from 18.5–23.9 kg/m^2^; Healthy body fat level was defined as a waist circumference of less than 90 cm. The above six types of lifestyle data were obtained from the self-reports of the participants. Each item that was met was counted as one healthy lifestyle, with a range of 0 to 6. The more items met, the healthier the lifestyle.

#### Multiple chronic conditions assessment

Based on the definition of multimorbidity and the findings from a survey conducted among hospitalized elderly Chinese patients with common chronic conditions, this study included six common chronic physical diseases: hypertension, diabetes, heart disease, chronic pulmonary disease, dyslipidemia, and cerebrovascular disease. All chronic diseases were diagnosed according to the International Classification of Diseases (ICD-10) standard (17).

#### Mental health assessment

The GAD-7, PHQ-9, and PSQI were used to assess common psychological problems such as anxiety, depression, and sleep disorders in the elderly. The GAD-7 ranges from 0 to 21 points, with a score of 10 or higher indicating the presence of anxiety symptoms. A score of 15 or more indicates severe anxiety. The higher the score, the more severe the anxiety. The PHQ-9 ranges from 0 to 27 points, with a score of 10 or above indicating depression. The higher the score, the more severe the depression. A score of 15 or more indicates severe depression (18, 19). The PSQI total score ranges from 0 to 21 points, with 19 items in total, assessing seven components of sleep, and a score above 7 indicates poor sleep quality (20).

### Quality control

All researchers received standardized training and successfully passed consistency tests. Clinical interviews, physical examinations, and evaluations were conducted by qualified clinical physicians. Blood samples must be drawn from the vein in the morning after an 8-h fast. After drinking enough water, the urinary system ultrasound was performed after the obvious desire to urinate. According to the Ellipse formula, prostate volume (ml) = 0.52 times height (cm) times length (cm) times width (cm) (21). After completion of this measurement, patients were instructed to urinate and a second sonogram of the bladder was performed to determine residual urine volume. Quality control personnel regularly review the quality of research data and exclude those who do not meet the standards. Disagreements were submitted to the research team for discussion and determination.

### Statistical analysis

All statistical analysis were performed using SPSS 24.0 statistical software (IBM Corporation, Armonk, NY, USA) and R (version 4.4.2). The Shapiro–Wilk test, p–p plot, and Q-Q plot were used to confirm normality for continuous variables. Normally distributed quantitative data were expressed as mean ± standard deviation, and group comparisons were conducted using the t test. Non-normally distributed quantitative data were expressed as M (Q1, Q3), and group comparisons were made using the Mann–Whitney U test. Count data were reported as categorical variables with numbers and group comparisons were carried out using Chi-square test. Pearson correlation coefficients were used to analyze the correlations between IPSS scores and clinical characteristics, lifestyle, multimorbidity, and mental health status. Binary Logistic Regression was used to analyze the effects of lifestyle, chronic physical disease and mental health on LUTS/BPH severity. A significance level of *p* < 0.05 was considered statistically significant.

## Results

### Sociodemographic and clinical characteristics of the participants

A total of 842 individuals completed the clinical interview and all examinations. Based on the evaluation results of the clinical interview and examinations, 806 valid samples were obtained after excluding individuals with severe anxiety and depression. The average age of the 806 effective samples was (71.47 ± 6.91) years old, while the average years of education was (10.63 ± 2.28) years. According to IPSS scores, the samples were divided into mild group with 218 cases and moderate-to-severe group with 588 cases. There were significant differences in age, disease duration, prostate volume (PV), total prostate specific antigen (tPSA), C-reactive protein (CRP), post-voided residual (PVR), and urea nitrogen (BUN) between the two groups (*p* < 0.05). However, no significant differences were found in marital status, education level, glomerular filtration rate, creatinine, and thyroid-stimulating hormone levels between the two groups (*p* > 0.05), as shown in Table 1.

**Table 1:** Comparison of sociodemographic and clinical characteristics between the two groups.

Variables	Total sample (*n* = 806)	Mild group (*n* = 218)	Moderate-to-severe group (*n* = 588)	*χ*^2^/*t*/*Z*	*p* value
Age, years	71.47 ± 6.91	68.40 ± 5.53	72.61 ± 7.02	8.90	<0.001
In marriage (yes/no)	618/188	166/52	452/136	0.05	0.829
Education level, years	10.63 ± 2.28	10.48 ± 2.29	10.69 ± 2.28	1.144	0.253
Duration of disease, months	21.99 ± 10.22	19.90 ± 9.05	22.76 ± 10.53	3.806	<0.001
PV, ml	50.42 ± 20.38	35.43 ± 10.90	55.98 ± 20.27	18.425	<0.001
tPSA, ng/ml	5.27(1.01, 6.19)	3.91(0.60, 5.28)	5.77(1.21, 6.52)	4.875	<0.001
CRP, mg/L	7.78(0.87, 7.96)	5.94(0.70, 5.00)	8.47(0.91, 8.85)	3.771	<0.001
PVR, ml	85.57(37.00, 83.00)	56.94(28.25, 72.00)	96.19(39.00, 100.00)	5.220	<0.001
GFR, ml/min	81.83 ± 15.99	83.01 ± 17.97	81.40 ± 15.19	1.274	0.203
BUN, mmol/L	6.19 ± 2.01	5.97 ± 1.62	6.28 ± 2.13	2.211	0.028
CREA, umol/L	82.62 ± 24.65	82.37 ± 22.15	82.71 ± 25.53	0.186	0.853
TSH, mIU/L	2.25(1.30,2.89)	2.26(1.23,2.86)	2.24(1.31,2.89)	0.690	0.490
T, nmol/L	7.14 ± 2.59	7.00 ± 2.60	7.19 ± 2.59	0.932	0.352
IPSS, score	14.12 ± 6.27	6.00 ± 1.32	17.12 ± 4.45	54.44	<0.001

Data are expressed as numbers; normally distributed data are shown as the mean ± SD; other continuous variables are shown as the median (interquartile range). Mann–Whitney U tests or *t* test were used to perform group comparisons, and Pearson’s *χ*^2^ test was used for categorical variables. *P* values < 0.05 were considered statistically significant. PV, prostate volume; tPSA, total prostate specific antigen; CRP, C-reactive protein; PVR, post voided residual; GFR, glomerular filtration rate; BUN, blood urea nitrogen; CREA, creatinine; TSH, thyroid stimulating hormone; T, testosterone; IPSS, international prostate symptom score.

### Lifestyle, multiple chronic conditions, and mental health status of the participants

Among the six categories of healthy lifestyle included in the analysis, the percentage of the total sample maintaining health was as follows: 70.72% for current non-smoking, 90.94% for non-excessive drinking, 58.93% for healthy eating habits, 47.02% for healthy weight, 25.68% for active physical exercise, and 36.72% for healthy body fat levels. The number of healthy lifestyle per capita in the total sample was (3.30 ± 1.23). The current smoking status, healthy eating habits, active physical exercise, healthy body fat percentage, and number of healthy lifestyle per capita exhibited a statistically significant disparity between the two groups (*p* < 0.01). The prevalence of chronic conditions in the total sample was as follows: hypertension 55.71%, heart disease 28.54%, dyslipidemia 23.33%, diabetes 22.95%, chronic lung disease 15.14%, and cerebrovascular disease 11.04%. The number of chronic conditions per capita in the total sample was (1.56 ± 1.01). Regarding mental health status, sleep disorders accounted for 34.74%, anxiety for 11.41%, and depression for 20.22% in the total sample population. Compared with the mild group, the moderate-to-severe group had significantly higher rates of hypertension, heart disease, dyslipidemia, diabetes, anxiety, depression, and sleep disorders (*p* < 0.05, Table 2).

**Table 2:** Comparison of lifestyle, multimorbidity and mental health status between the two groups.

Variables	Total sample (*n* = 806)	Mild group (*n* = 218)	Moderate-to-severe group (*n* = 588)	*χ*^2^/t	*P* value
Current non-smoking (yes/no)	570/236	174/44	396/192	11.942	0.001
Non-excessive drinking (yes/no)	733/73	195/23	538/50	0.809	0.368
Healthy diet (yes/no)	475/331	149/69	326/262	10.946	0.001
Healthy weight (yes/no)	379/427	112/106	267/321	2.274	0.132
Active exercise (yes/no)	207/599	78/140	129/459	15.963	<0.001
Healthy body fat (yes/no)	296/510	98/120	198/390	8.709	0.003
NHL, number	3.30 ± 1.23	3.70 ± 1.27	3.15 ± 1.25	5.464	<0.001
Hypertension (yes/no)	449/357	107/111	342/246	5.315	0.021
Heart disease (yes/no)	230/576	48/170	181/407	6.006	0.014
Dyslipidemia (yes/no)	188/618	35/183	153/435	8.831	0.003
Diabetes mellitus (yes/no)	185/621	33/185	152/436	10.321	0.001
Chronic lung disease (yes/no)	122/684	28/190	94/494	1.223	0.269
Cerebrovascular disease (yes/no)	89/717	17/201	72/516	3.201	0.074
NMCC, number	1.56 ± 1.01	1.23 ± 0.88	1.69 ± 1.03	6.287	<0.001
PHQ-9<10 (yes/no)	643/163	193/25	452/136	13.530	<0.001
GAD-7<10 (yes/no)	714/92	204/14	510/78	7.366	0.007
PSQI≤7 (yes/no)	526/280	164/54	362/226	13.099	<0.001

Data are expressed as numbers; normally distributed data are shown as the mean ± SD; *t* test was used to perform group comparisons, and Pearson’s χ2 test was used for categorical variables. *P* values < 0.05 were considered statistically significant. NHL, number of healthy lifestyles per capita; NMCC, number of multiple chronic conditions per capita; PHQ-9, patient health questionnaire-9; GAD-7, generalized anxiety disorder-7; PSQI, Pittsburgh sleep quality index.

### Correlation analyses between age, lifestyle, chronic conditions, mental health status and IPSS score

**Figure 1:**
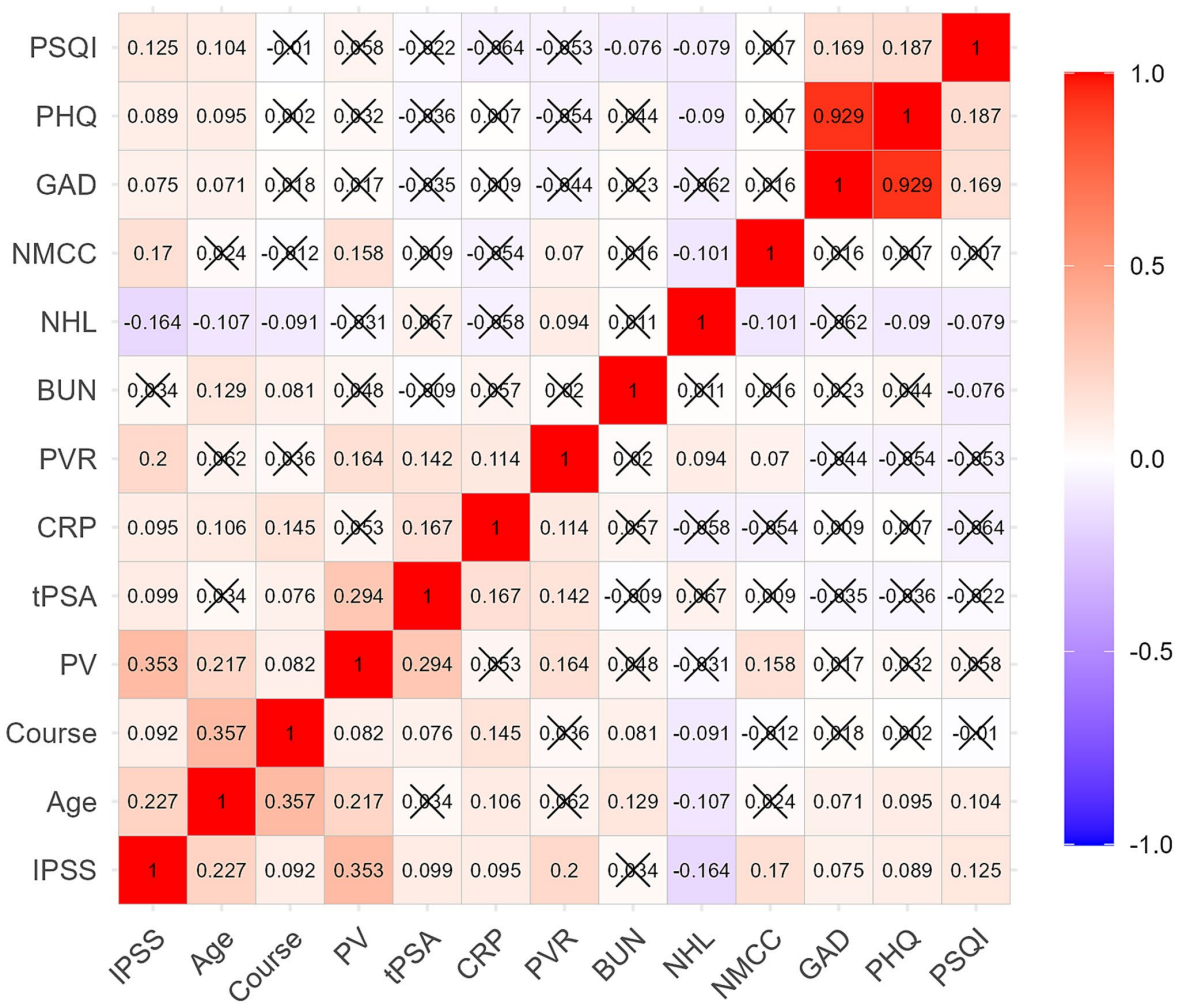
Correlation of IPSS score with age, clinical features, lifestyle, chronic conditions, and mental health status. The IPSS score was positively correlated with age, course, PV, tPSA, CRP, PVR, NMCC, PSQI, GAD-7, and PHQ-9 scores, and negatively correlated with NHL. Course: disease duration, NHL, number of healthy lifestyles per capita; NMCC, number of multiple chronic conditions per capita.

As shown in Figure 1, the IPSS score was positively correlated with age, disease duration, PV, tPSA, CRP, PVR, the number of chronic conditions per capita, PSQI, GAD-7, and PHQ-9 scores (*r* = 0.075–0.353, *P*<0.05), and negatively correlated with the number of healthy lifestyles per capita (*r* = −0.164, *P* < 0.05). Additionally, the number of healthy lifestyles per capita was negatively correlated with age, disease duration, the number of chronic conditions per capita, GAD-7, PHQ-9, and PSQI scores (*r* = 0.079–0.107, *P* < 0.05). The number of chronic conditions per capita was positively correlated with PV and PVR (*r* = 0.158, 0.070, *P* < 0.05).

### Association of LUTS severity with lifestyle, chronic conditions, and mental health status

**Figure 2:**
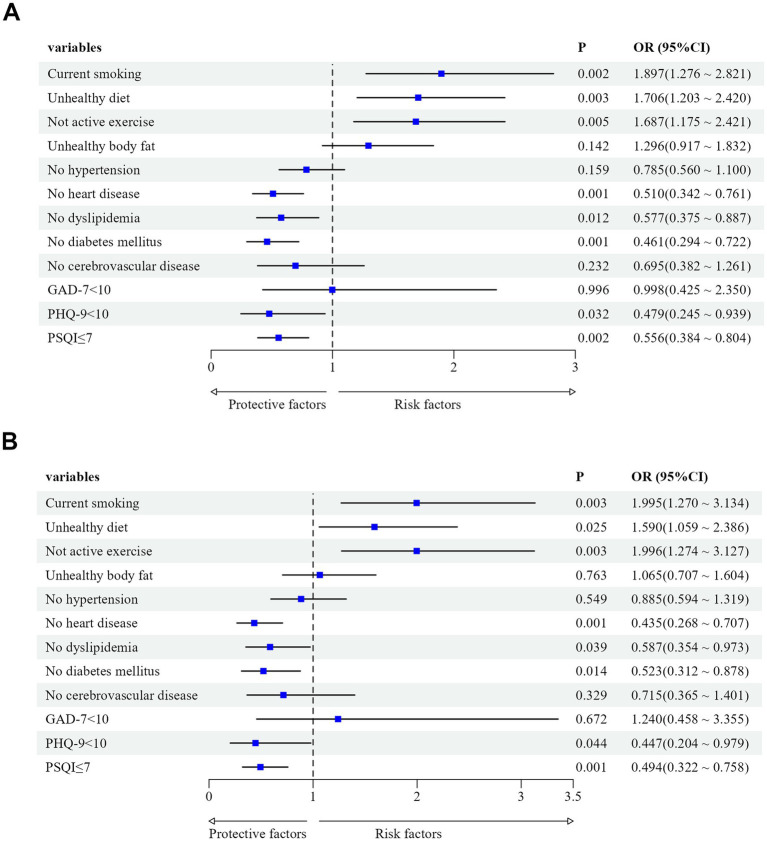
**(A)** Effects of lifestyle, chronic conditions, and mental health status on LUTS/BPH before adjusting for confounding factors. Confounding factors included age, disease duration, PV, tPSA, CRP, PVR, and BUN. **(B)** Effects of lifestyle, chronic conditions, and mental health status on LUTS/BPH after adjusting for age, disease duration, PV, tPSA, CRP, PVR, and BUN.

We utilized the degree of LUTS severity as the dependent variable (moderate/severe = 1, mild = 0) and included factors with *p* < 0.1 in univariate analysis as independent variables. Binary logistic regression analysis was employed to examine the data. The results revealed that current smoking, unhealthy dietary habits, and lack of physical activity were independent risk factors for the severity of LUTS/BPH, even after adjusting for age, disease duration, PV, tPSA, CRP, PVR, and BUN (*p* < 0.05, OR>1). Conversely, absence of diabetes or heart disease, normal lipid levels, and absence of depression or sleep disturbances were independent protective factors for the severity of LUTS/BPH (*p* < 0.05, OR<1) (Table 3; Figures 2A,B). Then, we performed the Hosmer-Lemeshow test on the adjusted regression model. The results indicated a satisfactory fit of the model, with *χ*^2^_h1_ = 12.166, df = 8 and *p* = 0.144.

**Table 3:** Logistic models of the associated factors of LUTS/BPH severity.

Variables	Model 1			Model 2		
	OR	95%CI	*P* value	OR	95% CI	*P* value
No current smoking						
Yes	1 (Ref)					
No	1.897	1.276–2.821	0.002	1.995	1.270–3.134	0.003
Healthy diet						
Yes	1 (Ref)					
No	1.706	1.203–2.420	0.003	1.590	1.059–2.386	0.025
Active exercise						
Yes	1 (Ref)					
No	1.687	1.175–2.421	0.005	1.996	1.274–3.127	0.003
Healthy body fat						
Yes	1 (Ref)					
No	1.296	0.917–1.832	0.142	1.065	0.707–1.604	0.763
Hypertension						
Yes	1 (Ref)					
No	0.785	0.560–1.100	0.159	0.885	0.594–1.319	0.549
Heart disease						
Yes	1 (Ref)					
No	0.510	0.342–0.761	0.001	0.435	0.268–0.707	0.001
Dyslipidemia						
Yes	1 (Ref)					
No	0.577	0.375–0.887	0.012	0.587	0.354–0.973	0.039
Diabetes mellitus						
Yes	1 (Ref)					
No	0.461	0.294–0.722	0.001	0.523	0.312–0.878	0.014
Cerebrovascular disease						
Yes	1 (Ref)					
No	0.695	0.382–1.261	0.232	0.715	0.365–1.401	0.329
GAD-7						
≥10	1 (Ref)					
<10	0.998	0.425–2.350	0.996	1.240	0.458–3.355	0.672
PHQ-9						
≥10	1 (Ref)					
<10	0.479	0.245–0.939	0.032	0.447	0.204–0.979	0.044
PSQI						
>7	1 (Ref)					
≤7	0.556	0.384–0.804	0.002	0.494	0.322–0.758	0.001
Age, years						
>75				1 (Ref)		
≤75				0.297	0.172–0.513	0.000
Duration of disease, months						
>20				1 (Ref)		
≤20				1.063	0.705–1.603	0.771
PV, ml						
>50				1 (Ref)		
≤50				0.102	0.059–0.176	0.000
tPSA, ng/ml						
>3.5				1 (Ref)		
≤3.5				0.614	0.403–0.933	0.022
CRP, mg/L						
>7.0				1 (Ref)		
≤7.0				0.197	0.114–0.339	0.000
PVR, ml						
>50				1 (Ref)		
≤50				0.535	0.359–0.798	0.002
BUN, mmol/L						
>7.2				1 (Ref)		
≤7.2				0.974	0.596–1.593	0.917

Model 1: crude model was not adjusted by any variables.

Model 2: adjusted for age, duration of disease, PV, tPSA, CRP, PVR, and BUN.

## Discussion

Although BPH is a non-fatal disease, its course is prolonged, easy to relapse, there is a lack of effective treatment, and seriously affect the health and quality of life of elderly males. According to statistics, LUTS/BPH-related healthy life lost years (YLDs) rank first among urological diseases, and the disease burden of BPH in China is increasing year by year. One of the reasons is the large number of elderly patients in China and the failure to effectively promote healthy behaviors, which has led to an increased risk of chronic diseases due to long-term exposure to unhealthy lifestyle behaviors. Therefore, implementing a range of measures to manage the reversible and modifiable risk factors of LUTS/BPH in order to prevent or delay disease progression has consistently been the primary focus of clinical attention. LUTS are the predominant reason for seeking treatment among BPH patients, and due to individual variations, a comprehensive assessment is essential prior to selecting treatment options. The IPSS is currently internationally recognized as the most effective tool for assessing the severity of BPH symptoms in patients (22). There have been studies showing that the severity of LUTS is related to educational level and living environment (23, 24). In our study, there was no statistical difference between the two groups in terms of marital status and education level, which may be related to the fact that more than half of the elderly people in Wuxi have received secondary school education. The effect of marital status on LUTS may also need to be combined with sexual life status to conduct high-quality analysis. The patients in the moderate-to-severe group were older, had longer disease duration, and higher PV, PVR, CRP, and BUN levels compared to those in the mild group. These findings are consistent with most domestic and international studies and align with the progression characteristics of BPH (25, 26). The levels of testosterone and thyroid-stimulating hormone did not show significant differences between the two groups. Sex hormones are known risk factors for the development of BPH, but the specific mechanism by which testosterone affects BPH remains unclear. It may be related to the saturation of androgen receptor (AR) and dihydrotestosterone (DHT) binding in the prostate (27). There is conflicting evidence regarding whether hyperthyroidism is a risk factor for BPH, requiring further research (28).

Lifestyle and healthy behaviors are widely recognized as factors that can affect the development of BPH. However, due to variations in economic conditions, geographical environments, and cultural backgrounds across different regions, there is considerable heterogeneity in the reported findings from related studies (5, 29). In our study, the total sample maintained an average of 3.12 healthy lifestyles per capita, comparable to the results reported by Zhu et al. (15). In the moderate-to-severe group, most dimensions of unhealthy lifestyle habits, the incidence rates of four common chronic diseases, and mental health problems were more severe than in the mild group. Although there was no difference in weight between the two groups, the probability of abnormal body fat was higher in the moderate-to-severe group than in the mild group. A longitudinal study found that as people age, they lose muscle tissue even if their weight is normal or decreasing, and the composition of body fat may also deviate from normal ranges (30). Parsons et al. (31) reported that obesity leads to immune cell infiltration, tissue remodeling, hyperplasia, and worsening of LUTS. The impact of alcohol consumption on the progression of BPH is inconclusive; most studies suggesting that moderate consumption of alcohol can lower plasma testosterone levels and have a preventive effect on BPH progression, while excessive alcohol consumption may damage the structural integrity of prostate cells and increase the risk of BPH (32, 33).

We found that the highest multimorbidity rate among all the samples was hypertension (55.71%), followed by heart disease (28.54%), dyslipidemia (23.33%), diabetes (22.95%), anxiety (11.41%), depression (20.22%) and sleep disorders (34.74%). These findings slightly deviated from previous domestic and international studies on this subject (34–37). Roehrborn et al. (34) reported that BPH/LUTS patients had comorbid hypertension, hyperlipidemia, and cardiovascular disease at rates of 53, 45, and 18%, respectively. Brock et al. reported that the incidences of hypertension, cardiovascular disease, and diabetes among BPH/LUTS patients in the United States and Europe were 43, 50, and 15%, respectively. Zhang et al. (36) reported that BPH/LUTS patients had depression at a rate of 29.1%, and Roehrborn et al. (34) reported that BPH/LUTS patients had anxiety/depression/sleep disorders at a rate of 16%. Additionally, Martin et al. reported a high incidence rate of poor sleep quality at approximately 32.2% among BPH/LUTS patients. Overall, the majority of BPH/LUTS patients in various countries or regions commonly exhibit comorbidities and mental health issues.

There have been studies indicating that the IPSS score does not show a correlation with residual urine volume or prostate volume. However, these findings were derived from European populations and did not consistently apply to Asian populations, highlighting the diverse clinical features of BPH across different racial groups (38, 39). We found that IPSS score was significantly correlated with age, disease duration, clinical characteristics, number of healthy lifestyle habits per capita, number of chronic diseases per capita, and level of mental health, and there was also a partial correlation between these factors. After adjusting for eight confounding factors related to demographics and clinical characteristics, we also found that the severity of BPH/LUTS was closely related to lifestyle, comorbidities, and mental health status. Among them, current smoking (*OR* = 1.995, 95%CI: 1.270–3.134), unhealthy dietary habits (*OR* = 1.590, 95%CI: 1.059–2.386), and lack of physical activity (*OR* = 1.996, 95%CI: 1.274–3.127) were positively correlated with the aggravation of LUTS/BPH; not having heart disease (*OR* = 0.435, 95%CI: 0.268–0.707), normal blood lipid levels (*OR* = 0.587, 95%CI: 0.354–0.973), no diabetes (*OR* = 0.523, 95%CI: 0.312–0.878), no depressive symptoms (*OR* = 0.447, 95%CI: 0.204–0.979), and no sleep disorders (*OR* = 0.494, 95%CI: 0.322–0.758) may reduce the risk of LUTS/BPH progression.

According to studies, smokers with BPH have higher levels of malondialdehyde and interleukin-8 in their blood, suggesting that smoking may promote the progression of BPH by inducing chronic inflammation in the prostate (40). Additionally, physical exercise can reduce the expression of androgen receptors and increase cell apoptosis in the prostate (41, 42), and aerobic exercise can also improve the expression of estrogen receptor *β* and alleviate BPH symptoms (43). Some evidence suggests that adhering to a Mediterranean diet has been linked to a reduced risk of BPH, whereas vitamin D deficiency can activate the NF-κB/IL-6 pathway and upregulate the stat3-mediated pathway leading to prostate inflammation and fibrosis. If a standard diet is restored, these effects can partially reverse (44). This suggests that health management, in addition to a balanced diet and avoiding smoking, should also include scientific physical activity intervention measures tailored to the specific needs of the elderly.

We found independent associations of diabetes, dyslipidemia, and cardiovascular diseases independently associated with LUTS. However, hypertension and cerebrovascular diseases showed no significant association with LUTS severity, which is consistent with previous literature reports but also differs in some aspects. Previous studies have shown that diabetes, hyperlipidemia, hypertension, and obesity are risk factors for BPH in the global population, but there may be differences between different ethnic groups. Several cross-sectional studies specifically targeting Asian populations have found that after controlling for confounding factors, type 2 diabetes and hypertension were not significantly associated with BPH (45). Although the impact of chronic somatic diseases on the development of BPH remains inconclusive, dyslipidemia, diabetes, heart disease are closely related to pro-inflammatory state, oxidative stress and pro-fibrotic conditions, and may share common pathophysiological pathways with LUTS. Several mechanisms can explain the association between multiple physical illnesses and BPH. Firstly, insulin can act on IGF receptors to promote prostate growth. Hyperinsulinemia and insulin growth factor can cause increased PV and PSA levels. Furthermore, insulin resistance has the potential to disrupt normal glucose regulation, impede systemic functions, and accelerate atherosclerosis progression, thus contributing to the development of BPH in individuals with comorbid diabetes. Lipids can induce the enlargement of adipocytes and the release of chemokines, and the chemokine IL-8 induces the expression of fibroblast growth factor (FGF-2), participates in the chronic inflammation associated with BPH, and mediates the proliferation of epithelial and stromal cells. The inflammatory mechanism may be an important defense mechanism for the body to restore homeostasis and tissue function, which exists in most non-communicable chronic diseases. It is speculated that multiple inflammatory factors such as IL-6a, IL1β, and TNF-*α* play a crucial role in BPH and cardiovascular diseases (46). Therefore, active control of multiple chronic diseases is of great significance for the prognosis of LUTS/BPH patients.

In addition to physical comorbidities, mental health is also associated with BPH/LUTS. Positive emotional states and quality sleep are favorable factors in delaying the progression of BPH/LUTS. A community-based study conducted by Zheng et al. demonstrated a significant positive correlation between poor sleep quality and LUTS in middle-aged and elderly Chinese men (OR: 2.486) (47). Additionally, Lee et al. (48) investigation in an Asian population revealed that increased severity of LUTS was associated with depressive tendencies. These findings align with the results of our study. Research conducted in European and American populations has similarly indicated that higher IPSS values are correlated with elevated Hamilton Rating Scale for Depression (HRSD) scores, suggesting that the relationship between LUTS and mental health may be independent of racial factors. The precise mechanism underlying the relationship between sleep disorders and LUTS remains incompletely understood. It may be associated with chronic inflammation, which could potentially play a role in the development of prostate-related diseases. Lack of sleep may promote the immune cascade (49, 50), and the transcription of inflammatory immune response genes is up-regulated, resulting in increased systemic inflammatory activity. Previous studies have demonstrated that insomnia patients exhibit elevated levels of IL-6, IL-10, IL-1β, TNF-*α*, CRP, and other inflammatory markers. The precise biological mechanism underlying the association between depression and LUTS remains elusive and may involve multiple biological pathways. Depressed individuals exhibit dysfunctional HPA axis activity, characterized by elevated plasma cortisol levels and decreased release of cortisol-releasing hormone (CRH). CRH, a neuropeptide responsible for coordinating the body’s overall stress response, exerts inhibitory effects on the brainstem-spinal cord pathway, thereby reducing the urination threshold and subsequent urine output. The relationship between depression and LUTS may run in both directions, with depressive symptoms more common in men with LUTS than in men without LUTS, depression independently causing LUTS, and stressful life events triggering LUTS (51, 52). Although psychological problems are more prevalent among the elderly population, non-psychiatric physicians have shown suboptimal rates of recognition. This study included psychological problems in the analysis, on the one hand, to help patients improve their prognosis, on the other hand, to attract the attention of urologists. LUTS should be managed from the perspective of bio-psycho-sociology rather than simply following the guidelines mechanically.

### Study strengths and limitations

In this study, the main advantage was that all cases of BPH were diagnosed based on prostate biopsy; secondly, we adjusted for multiple potential confounding factors; Thirdly, this study excluded patients with severe depressive or anxiety symptoms. Such patients are recommended to undergo further assessment and treatment in the psychological department. Given that such patients might require antidepressant medications, which carry the potential side effect of inducing urinary retention and could consequently interfere with the research outcomes, their exclusion was deemed necessary. Nevertheless, there were several limitations to the study. First, this study was conducted in a single urban hospital, and we cannot exclude the possibility of sample selection bias, which limits the general applicability of the research to other populations. Second, it was an observational study, so no definitive causal inferences can be drawn regarding the findings. Third, despite our efforts to incorporate a comprehensive set of observational variables, potential confounding factors may still exist and could potentially influence our results. Therefore, in the future, we need to conduct multicenter studies covering both urban and rural areas as well as patients from different regions across the country to enhance sample diversity and further verify the research results in different socio-cultural contexts.

## Conclusion

The results of this study suggest that the severity of BPH/LUTS is highly associated with lifestyle, comorbid chronic diseases, and mental health status. Therefore, treatment should not only focus on BPH itself but also establish a multi-disciplinary prevention and control alliance centered on hospitals and based in communities, cultivate patients’ proactive health behaviors, and develop individualized comprehensive treatment strategies.

## Data Availability

The raw data supporting the conclusions of this article will be made available by the authors, without undue reservation.
